# Linking Impulsivity to Activity Levels in Pre-Supplementary Motor Area during Sequential Gambling

**DOI:** 10.1523/JNEUROSCI.1287-22.2023

**Published:** 2023-02-22

**Authors:** Allan Lohse, Annemette Løkkegaard, Hartwig R. Siebner, David Meder

**Affiliations:** ^1^Danish Research Centre for Magnetic Resonance, Centre for Functional and Diagnostic Imaging and Research, Copenhagen University Hospital—Amager and Hvidovre, 2650 Hvidovre, Denmark; ^2^Department of Neurology, Copenhagen University Hospital Bispebjerg, 2400 Copenhagen, Denmark; ^3^Institute for Clinical Medicine, Faculty of Medical and Health Sciences, University of Copenhagen, DK-2200 Copenhagen, Denmark

**Keywords:** decision-making, fMRI, impulsivity, pre-supplementary motor area, risk, transcranial magnetic stimulation

## Abstract

Impulsivity refers to the tendency to act prematurely or without forethought, and excessive impulsivity is a key problem in many neuropsychiatric disorders. Since the pre-supplementary motor area (pre-SMA) has been implicated in inhibitory control, this region may also contribute to impulsivity. Here, we examined whether functional recruitment of pre-SMA may contribute to risky choice behavior (state impulsivity) during sequential gambling and its relation to self-reported trait impulsivity. To this end, we performed task-based functional MRI (fMRI) after low-frequency (1 Hz) repetitive transcranial magnetic stimulation (rTMS) of the pre-SMA. We expected low-frequency rTMS to modulate task-related engagement of the pre-SMA and, hereby, tune the tendency to make risky choices. Twenty-four healthy volunteers (12 females; age range, 19–52 years) received real or sham-rTMS on separate days in counterbalanced order. Thereafter, participants performed a sequential gambling task with concurrently increasing stakes and risk during whole-brain fMRI. In the sham-rTMS session, self-reported trait impulsivity scaled positively with state impulsivity (riskier choice behavior) during gambling. The higher the trait impulsivity, the lower was the task-related increase in pre-SMA activity with increasingly risky choices. Following real-rTMS, low-impulsivity participants increased their preference for risky choices, while the opposite was true for high-impulsivity participants, resulting in an overall decoupling of trait impulsivity and state impulsivity during gambling. This rTMS-induced behavioral shift was mirrored in the rTMS-induced change in pre-SMA activation. These results provide converging evidence for a causal link between the level of task-related pre-SMA activity and the propensity for impulsive risk-taking behavior in the context of sequential gambling.

**SIGNIFICANCE STATEMENT** Impulsivity is a personal trait characterized by a tendency to act prematurely or without forethought, and excessive impulsivity is a key problem in many neuropsychiatric disorders. Here we provide evidence that the pre-supplementary motor area (pre-SMA) is causally involved in implementing general impulsive tendencies (trait impulsivity) into actual behavior (state impulsivity). Participants’ self-reported impulsivity levels (trait impulsivity) were reflected in their choice behavior (state impulsivity) when involved in a sequential gambling task. This relationship was uncoupled after perturbing the pre-SMA with repetitive transcranial stimulation (rTMS). This effect was contingent on trait impulsivity and was echoed in rTMS-induced changes in pre-SMA activity. Pre-SMA is key in translating trait impulsivity into behavior, possibly by integrating prefrontal goals with corticostriatal motor control.

## Introduction

Impulsivity is a personal trait that describes one’s tendency to act prematurely or without forethought (Dalley and Robbins, 2017). Excessive impulsivity is intimately linked to a breakdown of inhibitory processes (Bari and Robbins, 2013) and constitutes a prominent clinical feature in many neuropsychiatric conditions (Kulacaoglu et al., 2017; Kozak et al., 2019). Trait impulsivity has multiple facets, commonly assessed with self-reports reflecting attentional, motor, and planning aspects of impulsivity (Stanford et al., 2009). Specific facets of impulsivity can also be experimentally quantified with tasks probing the inability to suppress premature response tendencies (“waiting impulsivity”; Voon et al., 2014), the failure to cancel already initiated responses (“stopping impulsivity”; Logan et al., 1984), or the preference for larger, less certain rewards over smaller, more certain rewards (“risky impulsivity”; Robbins and Dalley, 2017). While these tasks yield a momentary readout of the impulsive state of the individual at the time of examination (“state impulsivity”), behavioral measures of impulsivity often correlate only weakly with self-report trait measures of impulsivity and everyday risk-taking behavior (Schonberg et al., 2011; Cyders and Coskunpinar, 2012). Thus, there is little knowledge about whether and how trait impulsivity might mediate state impulsivity assessed in the laboratory.

Task-related functional MRI (fMRI) has been used extensively to identify brain networks implicated in impulsive behaviors. These studies have consistently reported altered cortical activity in the pre-supplementary motor area (pre-SMA) and right inferior frontal cortex (rIFG) in healthy individuals as well as patients experiencing psychiatric disorders with impaired impulse control (Taylor et al., 2007; Forstmann et al., 2008; Tabibnia et al., 2011; Ersche et al., 2012; Whelan et al., 2012). Together with electrophysiological studies, the fMRI studies corroborate the notion that the rIFG and pre-SMA, together with the subthalamic nucleus (STN), form a network supporting fast and nonselective suppression of inappropriate action tendencies, often referred to as the braking network (Aron et al., 2007; Wessel et al., 2019). Given the correlative nature of functional brain mapping, it is unclear whether and how the neural structures that have been associated with impulsivity are causally involved in the expression of impulsive actions.

Using task-related fMRI, we showed that the pre-SMA, IFG, and STN increase their activity during a sequential gambling task in which individuals either continue to gamble or stop in the context of increasing stake (Meder et al., 2016). These areas showed a gradual increase in activity with increasing stake, paralleled by a gradual increase in reaction time. Based on these findings, we argued that the braking network not only generates an acute stop signal to pause ongoing actions, but also implements a gradually increasing braking signal that prevents impulsive choices during sequential gambling. However, our fMRI findings are only correlational, and we did not test for a relationship between the variability in gambling behavior (state impulsivity) and the subject's self-reported trait impulsivity.

Following up on our fMRI study (Meder et al., 2016), we used a perturb-and-map approach to probe the causal contribution of the pre-SMA in controlling choice impulsivity during sequential gambling (Bergmann et al., 2016). We first targeted the pre-SMA with low-frequency (1 Hz) repetitive transcranial magnetic stimulation (rTMS) using robot-assisted neuronavigation. We then performed fMRI to probe the impact of rTMS on task-related activity in pre-SMA and risky choice behavior during gambling (state impulsivity), using the sequential gambling task introduced by Meder et al. (2016). We also assessed trait impulsivity using the Barratt impulsiveness scale (BIS-11).

We hypothesized that trait impulsivity would predict interindividual variations in the tuning of task-related activation of pre-SMA to gradual increases in stake as well as interindividual differences in choice behavior during the gambling task. We further predicted that the functional perturbation evoked by 1 Hz rTMS over the pre-SMA would modulate choice impulsivity expressed during the sequential task and that the modulatory effect of rTMS would depend on the participants’ trait impulsivity.

## Materials and Methods

### Participants.

We included 24 healthy adult volunteers (mean age, 28.2 years; SD, 9.7 years; age range, 19–52 years; 12 females) with no history of mental or neurologic illness. One participant was excluded from the analysis since this participant later revealed not having understood the task instructions for the first session, which was also clear from the change in the task performance between the two sessions. One participant was only included in the behavioral analysis since the data of one fMRI session was not saved because of an error.

The study was approved by the Research Ethics Committee of the Capital Region of Denmark (H-15017878), and all participants gave written informed consent before participating in the study.

### Experimental design.

**Figure 1.**
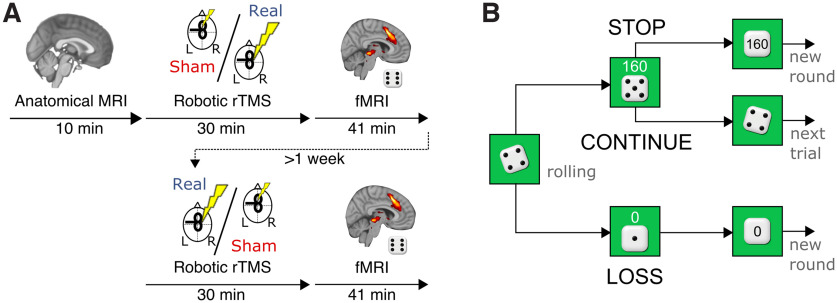
Task and experimental setup. ***A***, A total of 24 healthy volunteers participated in a 2 d study. On 2 d separated by at least 1 week, participants underwent 30 min of either inhibitory (1 Hz, 100% rMT) rTMS of the right pre-SMA or sham stimulation (right pre-SMA, 1 Hz, 30% rMT). The order of the stimulations was randomized and counterbalanced. Immediately following rTMS, participants performed a gambling task during fMRI recording to assess neural activity. ***B***, In the gambling task, participants accumulated points as they repeatedly rolled a die until they decided to bank the winnings and start a new round or until rolling a 1, resulting in the loss of all points in this round. Total winnings were the average of all rounds, including loss rounds.

The sequential gambling task was a computerized, open-ended, one-player version of the dice game “pig” first described by the American magician John Scarne (Scarne, 1945; Meder et al., 2016; Fig. 1). Each round began with a die being rolled automatically (the “rolling” was visualized by a rapid succession of one of the six sides of the die in random order; presentation time, 150 ms) for a jittered period of time (roll time, 1.5–3.5 s). Thereafter, the outcome of the throw was presented, and the participant had 2 s to decide to roll again and accumulate more points or to hold and bank the accumulated points. The participant could roll the die as many times as he or she wanted. The round ended when the participant held and thereby banked the sum of the rolls during that round or if he or she rolled a 1, in which case the accumulated sum was lost and a new round began. The outcome of the round was shown to the participant for 2.5 s. The payout of the game was the average sum accumulated in all rounds, including loss rounds, multiplied by 10 Danish kroner (DKK; 1 DKK = ∼$0.15 US). Every 6 min, participants switched between two types of task blocks that differed in terms of motor engagement. In “act-to-continue” blocks, participants had to actively press a button to continue with gambling and to refrain from a button press to end the gambling round to receive the accumulated sum. In “act-to-stop” blocks, sequential gambling did not require any action as the die was rolled automatically until participants actively ended the round by pressing a button. Between blocks, participants saw an instruction screen for 60 s. In this article, the different action contexts of gambling were not considered in the analysis. This aspect of the task will be addressed in a separate article.

The known, true odds for not losing every time a die is rolled are 5:1. The average winning amount is 40 DKK ( $\frac{2\;+\;3\;+\;4\;+\;5\;+\;6}{5}$ points * 10). Thus, if the accumulated sum is <200 DKK (resulting in odds of 200:40 to ∼5:1), the odds of winning are in favor of continuing rolling (Knizia, 2001). Thus, for a gain-maximizing strategy, participants should always stop the round once having accumulated ≥200 points.

### Repetitive transcranial magnetic stimulation.

Participants underwent real-rTMS [100% resting motor threshold (rMT)] and sham-rTMS (30% rMT) of pre-SMA in two separate sessions at least 1 week apart. The session order was counterbalanced across participants. For both sessions, over the course of 30 min 1800 biphasic pulses were applied over pre-SMA with a frequency of 1 Hz using a robot arm-controlled figure-eight coil (Ø, 2 × 75 mm; model MCF-B65, Medtronic). The coil was positioned in a lateral-to-medial orientation such that the strongest intracranial current went in a left-to-right direction, thus preferentially targeting the right hemisphere.

The individual resting motor threshold was determined in all sessions using the freeware TMS Motor Threshold Assessment Tool version 2.0 (MTAT 2.0; http://www.clinicalresearcher.org/software.htm), where the threshold is estimated using a maximum-likelihood strategy (“threshold hunting”). The rMT was assessed in the right hemisphere, and the motor evoked potential (MEP) of the left first dorsal interosseous was used as readout using the same coil type as the one used during the experiment. MEPs had to exceed 50 µV to be considered present. At the end of the stimulation, we asked participants to rate (on a scale of 1–10) “How effective do you feel the stimulation was?” (without explaining in further detail what was meant by “effective”) and “How uncomfortable did you find the stimulation?”

### Robot-assisted neuronavigation.

Right pre-SMA was localized using neuronavigation (LOCALITE) based on individual T1-weighted MRI scans acquired before the first TMS session. After normalizing the participants’ anatomic scans into standard MNI space, the stimulation target was set to [6, 16, 52] based on the peak activation from the previous study performed at our center using the same task (Meder et al., 2016). For the duration of the stimulation, the coil position was automatically maintained by a TMS robot (Axilum Robotics). After the stimulation, the participants were transferred to the scanner in an MR-compatible wheelchair.

### Structural and functional MRI.

Scans were acquired using a 3 T scanner (Verio, Siemens) with a 32-channel head coil. To relate the blood oxygenation level-dependent (BOLD) signal acquired with fMRI to anatomic brain structures and for rTMS neuronavigation a structural T1-weighted image was acquired [MPRAGE; repetition time (TR), 1900 ms; echo time (TE), 2.32 ms; flip angle, 9°; in-plane resolution, 0.89 × 0.89 mm; slice thickness, 0.9 mm; field of view (FOV), 256 mm). To assess task-related neural activity, regional BOLD signal changes were measured using a T2*-weighted EPI sequence (TR, 1650 ms; TE, 26 ms; flip angle, 74°). A total of 1459 volumes with 32 slices in ascending order were acquired per session (in-plane resolution, 3 × 3 mm; FOV, 192 mm). Axial slices were arranged parallel to the bicommissural line. Respiration and pulse were obtained with a pneumatic thoracic belt and pulse oximeter, respectively.

### Preprocessing of functional MRI data.

To allow for the T1 equilibrium effect, the first three volumes of each run were discarded. The EPI volumes of each session were slice time corrected, realigned to the first volume in the time series, and unwarped with the FSL topup tool. The unwarped images from the two sessions of each participant were then realigned to the mean image, segmented, coregistered to the segmented T1-weighted image, normalized to an MNI space template using affine warping and a discrete cosine transform basis (Ashburner and Friston, 1999), and smoothed with an 8 mm full-width at half-maximum Gaussian kernel. Volumes acquired during the pause between blocks were excluded (5 × 36 volumes per session).

### Analysis of functional MRI data.

The main aim of this study was to interrogate the causal involvement of the increase in pre-SMA activity during sequential gambling in the mediation of risky decision-making. Our main regressor of interest was thus the parametric modulation of continue events with the accumulated sum during that decision. The general linear model included the three main events of the paradigm [the presentation of the outcome of the dice roll, categorized into “continue,” “stop,” and “loss” trials, depending on the continue/stop decision made or the outcome being a “1” (loss)] and their parametric modulation with the accumulated sum in the trial in both block types (“act to continue” and “act to stop” blocks). Additional regressors of no interest were onsets of the dice roll, onsets of rounds where the first outcome was a 1, and onsets of the feedback screen for both loss and win rounds. Heart and pulse rates were added to the design matrix as nuisance regressors (Lund et al., 2006). Region of interest (ROI) analyses were performed using a pre-SMA mask defined as a sphere (*r* = 5 mm) centered at the stimulation target (MNI coordinates *x*, *y*, *z* = 6, 16, 52), putamen using the anatomic masks from WFU_pickAtlas (Maldjian et al., 2003), and subthalamic nucleus using a probabilistic mask based on 7 T MRI (Keuken et al., 2014).

Preprocessing and analysis of the images were performed in SPM12 (revision 6906, Wellcome Department of Imaging Neuroscience, Institute of Neurology, University College London).

### Statistical analysis.

Statistical analyses of behavior were performed in R (R Foundation for Statistical Computing). We used a linear mixed model implemented in the nlme package (see https://CRAN.R-project.org/package=nlme) with regressors motor context (act to continue, act to stop), rTMS condition (real-rTMS, sham-rTMS), and session number (session 1, session 2) for the analysis of mean stopping amounts.

Response times (RTs) were log-transformed to meet the assumption of normal distribution. The mean ± SD is reported. For correlational analyses, Pearson’s *r* is reported. To test whether participants might have been aware of differences between real and sham stimulation, we used Bayesian paired *t* tests to test for differences between their ratings of stimulation effectiveness and discomfort (JASP software version 0.14.3). We used Bayesian tests to be able to test for the absence of an effect. Bayes factors are classified according to the scheme of Jeffreys [1998; Bayes Factor (BF) = 1–3, anecdotal evidence; BF = 3–10, moderate evidence; BF = 10–30, strong evidence; BF = 30–100, very strong evidence; BF > 100, extreme evidence]. BF_10_ denotes evidence in favor of a given model against the null model, while BF_01_ denotes evidence in favor of the null model (Keysers et al., 2020).

## Results

### Effects of real-rTMS on choice behavior

**Figure 2.**
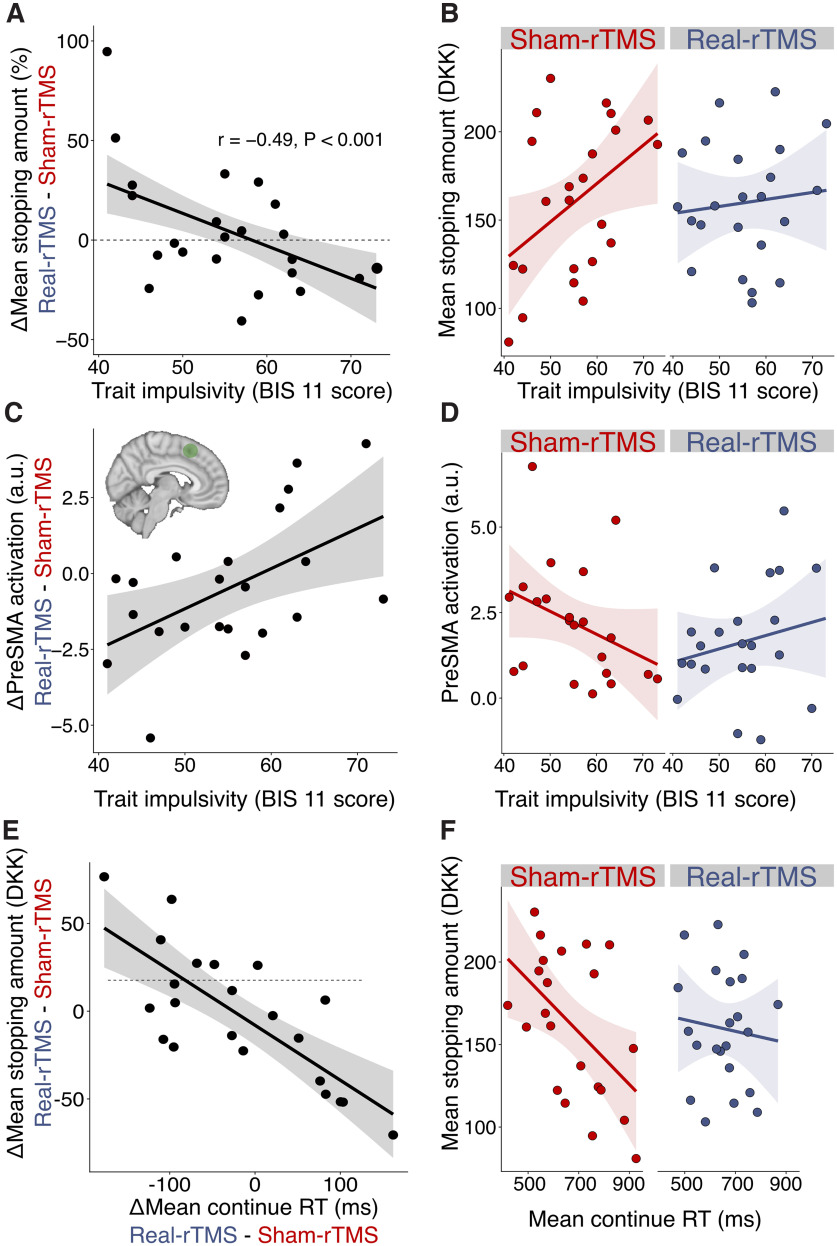
Pre-SMA causally modulates impulsivity during continue-to-gamble decisions. ***A***, Trait impulsivity predicts rTMS effect on the mean stopping amount. Participants who reported low degrees of impulsivity increased their tendency to make risky choices, whereas the opposite was true for those who reported high levels of impulsivity. ***B***, The 1 Hz real-rTMS decouples the association between trait impulsivity and task-related risk-taking behavior. In the sham-rTMS condition, impulsivity, measured using a standard inventory (BIS-11), correlated with the mean stopping amount in the dice game. Inhibiting the pre-SMA with 1 Hz rTMS decorrelated self-reported trait impulsivity and decisional impulsivity during the task. ***C***, Trait impulsivity predicts the rTMS effect on overall task-related brain activation during continue trials. A real-rTMS of 1 Hz lowers the linear increase in pre-SMA BOLD signal parametrically modulated by the continue sum and covarying with self-reported trait impulsivity. The ROI was defined as a sphere (*r* = 5 mm) centered at the stimulation target (MNI coordinates *x*, *y*, *z* = 6, 16, 52). a.u. = arbitrary units. ***D***, Visualization of the rTMS effect on the change of pre-SMA activation for the two sessions separately. ***E***, Association between the rTMS-induced change in mean continue response times and the rTMS-induced change in mean stopping amounts. The more rTMS slows down response times during continue decisions, the earlier the amount at which participants decide to stop a round. ***F***, Visualization of the rTMS effect on the change in mean stopping amount for the two sessions separately. The 1 Hz real-rTMS decouples the association between mean continue response times and mean stopping amount. *N *=* *23 participants.

Participants completed a mean total of 528.8 continue trials, 135.2 stop trials, and 121.6 loss trials. Real-rTMS did not change the mean stopping amount significantly compared with sham-rTMS (mean sham-rTMS, 160.40; mean real-rTMS, 159.81; paired *t* test: *t*_(22)_ = 0.08, *p* = 0.94). However, in the sham-rTMS session, there was a significant correlation between self-reported impulsivity (BIS-11 score) and mean stopping amount (*r *=* *0.43, *p *=* *0.040). The higher the individual BIS score, the larger was the mean amount at which participants decided to stop the round and bank their accumulated earnings (mean stopping amount). This relationship between self-reported trait impulsivity, indexed by the BIS-11 score, and state impulsivity, expressed by the participant’s choice behavior during sequential gambling (mean stopping amount), was no longer present after real-rTMS (*r *=* *0.15, *p* =0.48). Accordingly, we found a significant interaction between self-reported impulsivity and rTMS condition for the mean stopping amount (*p *= 0.017; Fig. 2*B*). The relative effect of real-rTMS on mean stopping amount was inversely related to the participants’ trait impulsivity (*r* = −0.49, *p *>* *0.001; Fig. 2*A*): the higher the participant’s trait impulsivity, the more did real-rTMS reduce state impulsivity (stopping behavior) relative to sham-rTMS. Conversely, the lower the participant’s trait impulsivity, the more did real-rTMS increase task-related state impulsivity relative to sham-rTMS. Together, these findings indicate that real-rTMS over pre-SMA led to a decoupling of trait impulsivity and state impulsivity.

We unfortunately were unable to recover the ratings of stimulation effectiveness and discomfort of six participants. Analyzing the remaining 16 datasets, we found that both effectiveness and discomfort were rated lower in the sham-rTMS condition compared with the real-rTMS condition (mean rated effectiveness: real-rTMS, 4.63; sham-rTMS, 3.19; mean rated discomfort: real-rTMS, 3.49; sham-rTMS, 2.31). However, this effect was small, and the Bayes factors showed anecdotal evidence in favor of the absence of a difference (BF_01_ effectiveness, 1.72; BF_01_ discomfort, 1.52).

### Response times

Decisions to continue were made more slowly as the accumulated sum increased after both sham-rTMS (mean slope of log-transformed continue RTs, 0.00174; SD, 0.00193; one-sample *t* test: *t*_(22)_ = 4.3352, *p *=* *0.000266) and real-rTMS of the pre-SMA (mean slope, 0.00172; SD, 0.00,152; one-sample *t* test: *t*_(22)_ = 5.418, *p *<* *0.0001), but there was no difference between the two rTMS conditions (paired *t* test: *t*_(22)_ = 0.075978, *p *=* *0.9401). Conversely, decisions to stop gambling and bank the winnings were made more quickly when the stake became higher in both the sham-rTMS (mean slope of log-transformed stop RTs, −0.0025; SD, 0.0026; one-sample *t* test: *t*_(22)_ = −4.6303, *p *=* *0.0001), and real-rTMS session (mean slope, −0.0019; SD, 0.002; one-sample *t*_(22)_ = −4.5476, *p *=* *0.0002), but there was no significant difference between the two rTMS conditions (paired *t* test: *t*_(22)_ = 1.09, *p *=* *0.29). We also tested whether there might be individually specific rTMS effects on this slowing/speeding depending on their state impulsivity. We did not find a correlation between the rTMS effect on the amount-induced slowing of reaction times for continue decisions and the rTMS effect on the mean stopping amount (Pearson’s *r* = −0.131, *p* = 0.561). There was no significant correlation between the amount-induced speeding up of reaction times stop decisions and the rTMS effect on mean stopping amount either (Pearson’s *r* = 0.040, *p* = 0.859).

However, there was a clear association between the rTMS-induced change in mean continue response times (not their slowing or speeding) and the rTMS-induced change in mean stopping amounts (*r* = −0.764, *p* < 0.001; Fig. 2*E*,*F*). There was no such correlation between the TMS effect on mean stopping amount and the TMS effect on response times during stop decisions (Pearson’s *r* = 0.001, *p* = 0.969).

### Functional magnetic resonance brain imaging

**Figure 3.**
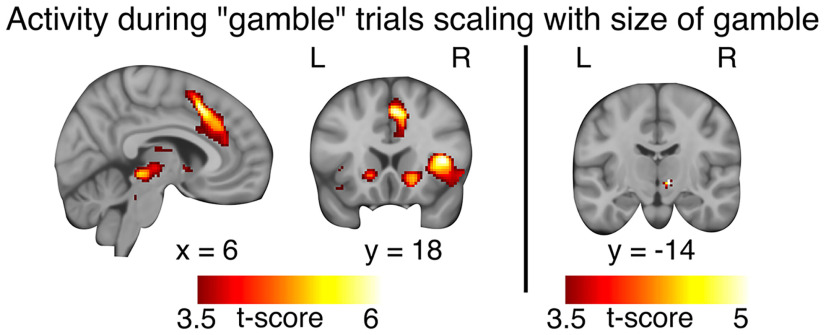
Brain regions that showed an increase in activity with the accumulated sum during continue trials (Table 1). *N *=* *23.

In the sham-rTMS session, a midline cluster showed a linear increase in “continue-to-gamble” activity with increasingly higher stakes, comprising the pre-SMA and dorsal anterior cingulate cortex (ACC; Fig. 3, Table 1). The ventral and dorsal striatum, anterior insula, lateral intraparietal cortex, inferior parietal lobule, right inferior frontal cortex, subthalamic nucleus, and occipital cortex also showed an increase in continue-to-gamble activity during a sequential gambling round that scaled with the accumulated sum (Fig. 3, Table 1), replicating the activity pattern reported in our previous study (Meder et al., 2016).

**Table 1:** Significantly activated clusters during sequential gambling

Region	*z* score peak	Peak MNI coordinates: left hemisphere			*z* score peak	Peak MNI coordinates: right hemisphere		
		*x*	*y*	*z*		*x*	*y*	*z*
Sham-rTMS session only								
Task-related activity during continue trials showing a linear increase with cumulative gamble sum						
Occipital cortex (C1)	5.09	−20	−94	−8			
Anterior insula (C2)					4.92	38	20	6
Ventral Striatum (C2)					4.65	12	10	−4
Pre-supplementary motor area (C3)					4.81	4	20	44
Pre-supplementary motor area/rostral cingulate zone (C3)					4.19	8	30	30
Dorsal anterior cingulate cortex (C3)					4.11	8	36	20
Inferior parietal lobule (C4)					4.52	34	−74	30
Lateral intraparietal cortex (C4)					4.46	40	−40	44
Inferior frontal cortex (C5)					4.46	34	4	28
Occipital cortex (C6)					4.29	30	−90	4
Subthalamic nucleus (C7; SVC)					4.29	10	−14	−4
Ventral striatum (C8)	4.08	−14	16	−4			
Putamen (C8; SVC)	3.89	30	18	0			
Real-rTMS > sham-rTMS								
Task-related activity during continue trials showing a larger linear increase with cumulative gamble sum after real-rTMS > sham-rTMS covarying with change in mean stopping amount						
Dorsal anterior cingulate cortex	4.13	−6	8	32			

Significant activation peaks (*z* score) and the corresponding stereotactic *x*, *y*, and *z* coordinates in MNI space from the standard model. Clusters are defined at *p* < 0.001, uncorrected, at whole-brain level (*t* > 3.579), cluster extent threshold of 30 voxels, cluster-level *p* < 0.05 FWE corrected. C, Cluster; SVC, small volume correction.

The effect of real-rTMS over pre-SMA on choice behavior also had a functional correlate in the stimulated pre-SMA. The rTMS-induced change in activity in the stimulated pre-SMA was related to self-reported trait impulsivity (Fig. 2*C*,*D*). The higher the participant’s BIS-11 score, the more did real-rTMS steepen the increase in pre-SMA activity with the size of the gamble (Fig. 2*C*). The lower the participant’s BIS-11 score, the more real-rTMS flattened the rise in pre-SMA activity with the size of the gamble (Fig. 2*C*). This led to a reversal of the relationship between the BIS-11 score and pre-SMA activation with increasing gamble size after real-rTMS relative to the sham-rTMS session (Fig. 2*D*).

## Discussion

Here we used a perturb-and-map approach to test for a causal link among task-related activity in pre-SMA, impulsive choice behavior during a sequential gambling task (state impulsivity), and self-reported trait impulsivity. Using robot-assisted 1 Hz rTMS, we first induced a perturbation of pre-SMA or performed sham stimulation. We then performed fMRI to map how real-rTMS altered regional activity in pre-SMA and impulsive choice behavior relative to sham-rTMS. We were particularly interested in assessing whether real-rTMS altered the scaling of continue-to-gamble activity in pre-SMA to the accumulated sum of reward during a sequential gambling round.

Our study yielded several main findings. In the unperturbed (sham-rTMS) state, self-reported trait impulsivity showed a positive linear relationship with state impulsivity (risky impulsivity; Robbins and Dalley, 2017), reflected by the inclination to continue gambling for higher rewards under increasing risk. Trait impulsivity was also reflected in the task-related activation profile of pre-SMA in the sham-rTMS session. The lower the individual BIS-11 score, the more the pre-SMA increased its activity with increasing stake. Real 1 Hz-rTMS of pre-SMA abolished these relations, and the perturbation effects of real-rTMS on choice behavior and pre-SMA activity differed among individuals depending on their trait impulsivity. In the following, we first discuss the link between task-related pre-SMA activity and impulsive choice behavior in relation to the suggested role of the pre-SMA as a “brake.” We then discuss the implications of how the perturbation of pre-SMA affected the relationship between trait and state impulsivity as well as pre-SMA activity.

### Does the pre-SMA function as a brake to reduce impulsive behavior?

The increase in pre-SMA activation at higher levels of risk and rewards replicates our own previous study (Meder et al., 2016) and is in line with other studies with similar tasks, including the balloon analog risk-taking task (Rao et al., 2008; Helfinstein et al., 2014). Together with the pre-SMA, all key regions in the braking network (i.e., inferior frontal cortex, striatum, and subthalamic nucleus) increase linearly with increasing stakes during continue trials. This network has been implicated in stopping behavior and conflict management (Jahfari et al., 2010; Aron et al., 2014, 2016). In our previous study, we found that participants with more cautious gambling behavior in the task had a stronger STN–pre-SMA coupling than participants who took more risky decisions, and we thus previously interpreted the gradual buildup in activity and connectivity in this network as an increasing cognitive brake or caution with higher sums at stake (Meder et al., 2016). However, the fact that this increase in reaction times with increasing stakes was not altered by rTMS perturbation of the pre-SMA argues against a simple “braking” function. We then asked whether the braking function might be differentially affected by rTMS, depending on each participant’s individual impulsivity, but we did not find any such association either. Our results thus suggest that, at least in this experimental setting, the pre-SMA might not be directly involved in the dynamic response slowing over the course of a sequence of increasingly risky choices as we had previously assumed. We did, however, find that perturbation of the pre-SMA with rTMS had an effect on average response times that was dependent on the individual participant’s impulsivity. In the unperturbed state, more cautious behavior (stopping the rounds at lower amounts) was associated with longer reaction times. Interestingly, taking a longer time for deliberation did not optimize decisions in terms of gain maximization as the more cautious behavior led to stopping decisions at amounts far below the optimal 200 kroner. Inhibitory rTMS over the pre-SMA did not have a general effect on mean reaction times but was dependent on the state impulsivity. In participants where rTMS reduced stopping amounts, this was related to an increase in mean reaction time, and vice versa. Thus, we find no effect of pre-SMA stimulation on the response slowing (braking) for riskier decisions, but instead observe that stimulation abolishes the association between caution reflected in response times and caution in terms of stopping amounts. This supports the idea that the pre-SMA might be less involved in the inhibitory aspect of response control, but rather more in motivational aspects of cognitive control in general (Ridderinkhof et al., 2004; Shenhav et al., 2013; Cavanagh and Frank, 2014) and sequential value-based decision-making under risk in particular (Schonberg et al., 2012; Pearson et al., 2014).

### Relationship between trait and state impulsivity

Another question we wanted to address in our experiment was whether the pre-SMA plays a role in the relationship between trait and state impulsivity. In the sham-rTMS session, self-reported trait impulsivity, as indexed by the BIS-11 score, scaled positively with risky impulsivity during the sequential gambling task (i.e., with state impulsivity probed by the task context). This is in contrast to many other studies where behavioral measures of impulsivity, including measures of stopping impulsivity, waiting impulsivity, and reflection impulsivity as well as tasks applying economic notions of risk oftentimes do not reflect underlying trait impulsivity or risky behaviors in life (Cyders and Coskunpinar, 2011; Schonberg et al., 2011). While most experiments attempt to decouple different variables to be able to unambiguously interpret their effects on outcome measures, in this paradigm, risk and reward are coupled. This coupling is a feature of many risky decisions in real life, and thus our task might elicit more naturalistic risk-taking behavior, allowing an association with trait measures of impulsivity to emerge (Schonberg et al., 2011).

Task-related fMRI showed a link between task-related activity in the pre-SMA and self-reported trait impulsivity. The higher the self-reported trait impulsivity, the smaller was the task-related increase in pre-SMA activity with increasingly risky choices.

Perturbing the pre-SMA with real-rTMS decoupled the association between trait and state impulsivity. Participants with low trait impulsivity increased their preference for risky choices after real-rTMS of pre-SMA, while the opposite was the case for participants with high trait impulsivity. These individuals showed a reduction in risk-taking behavior during gambling after real-rTMS.

The rTMS-induced behavioral shift was mirrored by an rTMS-induced change in task-related activity in the pre-SMA target. In individuals with high trait impulsivity, real-rTMS increased the otherwise low scaling of pre-SMA activity with the accumulated sum, whereas it decreased the otherwise higher scaling of activity in individuals with low trait impulsivity. As with rTMS-induced decoupling of mean response times and stopping amounts, inhibiting the pre-SMA had an equalizing effect on both the relation between state and trait impulsivity (i.e., more risk seeking behavior in low impulsivity individuals, less risk seeking in high impulsivity individuals) and between trait impulsivity and task-related pre-SMA activity (i.e., weaker activity increase with increasing stakes in low-impulsivity individuals, stronger activity increase in high-impulsivity individuals).

Pre-SMA is densely connected with the dorsolateral prefrontal cortex (dlPFC) and the more caudal SMA proper. One theory, based primarily on fMRI data, states that cognitive control of behavior is, at least in part, controlled by the prefrontal cortex organized hierarchically in a rostrocaudal order with the dlPFC on top and the cortical motor system at the bottom of the hierarchy (Badre and Nee, 2018). The pre-SMA is at the interface between higher-order prefrontal areas and lower-order motor areas. It has strong connections to dlPFC and the lower bank of ACC (Luppino et al., 1993; Lu et al., 1994) and contains few corticospinal neurons (Luppino et al., 1994). It is, however, tightly coupled to the SMA proper and still considered a motor area (Rizzolatti and Luppino, 2001). Together with its direct connectivity with key inhibitory control regions [IFG (Luppino et al., 1993) and STN (Nambu et al., 1996)], this suggests that the pre-SMA might play an important role in translating strategic goals and long-term behavioral biases (traits) from prefrontal areas into specific adaptive actions implemented by the motor system. We speculate that by perturbing the activity in pre-SMA the rostrocaudal chain of information might be impaired, leading to a decoupling of long-term, abstract goals, represented in the more rostrolateral prefrontal cortex and the motor implementation in the cortical motor system. This might lead to the decoupling of trait impulsivity and state impulsivity (i.e., the task-related choice behavior).

This baseline dependency is an effect oftentimes observed in the context of neuromodulatory neurotransmitters such as dopamine or noradrenaline, where too low and too high levels of the neurotransmitter lead to poorer task performance (Robbins and Arnsten, 2009; Dayan, 2012; Meder et al., 2019). It is often referred to as the Yerkes–Dodson function or an inverted U-shape (Robbins, 2000). Perturbing the pre-SMA might release the region from the effects of prefrontally mediated “too low” and “too high” levels of trait impulsivity that otherwise modulate the response of the area to risky decisions and the resulting choice behavior. Personality traits might be associated with different baseline levels of neural activity in the pre-SMA, which may result in bidirectional effects of rTMS depending on the level of activity such that high activity level leads to suppression and low activity level leads to the facilitation of neural activity reflecting the principle of homeostatic plasticity (Siebner et al., 2004). This moves activity and behavior toward the highest point of the inverted U-shape. Note, however, that real-rTMS did not actually lead to more optimal stopping behavior in the sense of gain maximization (i.e., the mean stop amount was not closer to the gain-maximizing 200 DKK). Optimal behavior should here thus be understood in terms of utility where risk and loss aversion are commonly observed in gambling decisions (Kahneman and Tversky, 1979).

It should be noted that because of data loss on the computer used for neuronavigation (TMS coil placement), we were unable to perform field modeling of the induced rTMS-induced e-field. We thus cannot exclude the possibility that rTMS disrupted signaling in other brain regions such as the dorsal ACC, which also plays an important role in sequential decision-making (Hayden et al., 2011; Kolling et al., 2014).

Another possible confound might have been that participants would have noted a difference between the real and sham TMS stimulation, but we found no evidence for this. Furthermore, even if participants had formed some idea about the stimulation they received, we deem it unlikely to have affected their behavior in any consistent manner. First, they most likely would not know about the potential effects of TMS (or whether the stimulation was excitatory or inhibitory). Second, even if they had some idea about a difference in stimulation effect, we find it difficult to conceive how this idea (and not the actual stimulation effect itself) should have led to consistent changes in behavior that would have affected our results.

To our knowledge, this is the first study to show that pre-SMA is centrally involved in translating trait impulsivity into actual choice behavior. Furthermore, we provide evidence that the functional significance of pre-SMA activity for choice behavior under risk depends on the trait impulsivity of the individual. In future studies, perturbation of pre-SMA in populations with excessive levels of impulsivity such as pathologic gambling or substance abuse disorder could help gain mechanistic insight and potential treatment options for these complex and disabling disorders.
